# SMS reminders to improve adherence and cure of tuberculosis patients in Cameroon (TB-SMS Cameroon): a randomised controlled trial

**DOI:** 10.1186/s12889-018-5502-x

**Published:** 2018-05-02

**Authors:** Georges Bediang, Beat Stoll, Nadia Elia, Jean-Louis Abena, Antoine Geissbuhler

**Affiliations:** 1 Faculty of Medicine and Biomedical Sciences, University of Yaoundé I, P.O Box: 1364, Yaoundé, Cameroon; 2 Geneva Tumour Registry, Institute of Global Health, Faculty of Medicine, University of Geneva, Geneva, Switzerland; 3 National Tuberculosis Control Program, Ministry of Public Health, Yaoundé, Cameroon; 4 Department of Radiology and Medical Informatics, Faculty of Medicine, University of Geneva, Geneva, Switzerland

**Keywords:** SMS-reminders, Text messaging, mHealth, Effectiveness, Tuberculosis, Developing countries, Low-middle income countries, Africa

## Abstract

**Background:**

In Cameroon, the National Tuberculosis Control Program that applies selective directly observed treatments faces difficulties in its implementation for a lack of resources, leading to only 65% of patients with sputum smear-positive pulmonary tuberculosis being cured after 6 months of treatment. This study aimed to evaluate the effectiveness of daily Short Message Service reminders to increase adherence and the proportion of adult tuberculosis patients cured after 6 months of treatment.

**Methods:**

A simple blinded, randomised controlled, multicentre study carried out in 12 Treatment and Diagnostic Centres of Yaoundé. The patients included were randomly assigned to two groups: patients in the intervention group received daily SMS reminders in addition to the usual treatment; those in the control group received the usual treatment only. The primary outcomes were the number and proportion of treatment success at 5 months, and the number and proportion of patients cured at 6 months. Data analysis was by intention to treat.

**Results:**

Two hundred and seventy-nine participants were randomized into intervention group (*n* = 137) and control group (*n* = 142). At five months, there were 111 treatment success (81%) in the intervention group and 106 (74.6%) in the control group (OR = 1.45 [0.81, 2.56]; *p* = 0.203). At 6 months, there were 87 patients cured (63.5%) in the intervention group and 88 (62%) in the control group (OR = 1.06 [0.65, 1.73]; *p* = 0.791). The number of drop-outs at 6 months was 47 (34.3%) in intervention group, and 46 (32.4%) in the control group. 48.9% (23/47) and 39.1% (18/46) of these drop-outs were sputum-negative at 5 months. At three different appointments, there were no significant differences between the two groups in any secondary outcomes. Very high and similar satisfaction was found for general management of patients in both groups: 99.5 and 99.2% (*p* = 0.41).

**Conclusions:**

Our study suggests that SMS reminders do not increase treatment success and cure proportions. However, the low proportion of patients cured at 6 month may be an underestimation due to a high dropout rate between the fifth and the sixth months of treatment. Future trials should focus on reducing the dropout rate.

**Trial registration:**

The trial was registered on the Pan-African Clinical Trials Registry (PACTR201307000583416 of 22 July 2013) and the protocol was published.

## Background

Tuberculosis (TB) is one of the major public health problems in developing countries [1, 2]. In Cameroon, the prevalence of tuberculosis (all forms included) is of 122 per 100,000 inhabitants. In 2010, 24,528 cases (all forms included) were recorded. The number of new cases of sputum Koch smear positive pulmonary tuberculosis was 14,464 [3]. The proportion of co-infection with HIV was estimated at 33% [3].

To address TB problem around the world, the international community led by the World Health Organisation (WHO) and its partners, developed international strategies and guidelines to fight against TB: Directly Observed Treatment Short Course (DOTS [4]), Global Strategy and Plan to Stop TB ([5, 6]) and now, WHO End TB Strategy [7].

The DOTS strategy was designed to improve the management of TB patients in order to increase their adherence to the full treatment of 6 months, and the proportion of cured patients. Failure to comply with the 6 months leads to high risk for relapse and increases overall resistance against the anti-TB drugs. This strategy includes a sustained political commitment; access to quality-assured TB sputum microscopy; standardized short-course chemotherapy to all cases of TB under proper case-management conditions including direct observation of treatment (DOT); uninterrupted supply of quality-assured drugs; and recording and reporting system enabling outcome assessment [8].

Because of numerous infrastructural, organizational, economic, and logistic limits in developing countries [9–11], the overall DOTS strategy is not correctly implemented and adaptations were developed. For example, direct observation of patient taking the medication by a healthcare worker during the intensive treatment phase is rarely correctly implemented. All these limiting factors lead to a reduction of the effectiveness of the program, namely poor adherence of patients to treatment and its consequences such as low cure proportion, relapse, or drug resistance. [12–16]

In Cameroon, the DOTS strategy is implemented by the National Program for the fight against tuberculosis (PNLT) [3] financed by the Government of Cameroon in collaboration with international funding partners such as The Global Fund to Fight AIDS, Tuberculosis and Malaria. Instead of receiving daily directly observed drug intake during the first 2 months of treatment, the patient receives several doses on a weekly or monthly basis. In 2010, only around 77% of sputum smear-positive pulmonary tuberculosis (SS + PTB) patients completed their 6 months treatment, with confirmed sputum smear negativity at 5 months and 65% were considered “cured” (patients who completed 6 months treatment with a sputum smear-negative result at 6 months) [17]. This proportion of cure is 20% below the WHO objective [8]. The main cause of low cure proportion in Cameroon is non-adherence to treatment of patients [3]. This could be due to lack of communication and interactions between healthcare professionals and patients through the care process.

Based on the progressive dissemination of mobile health technology (mHealth) and its feasibility, acceptability and ability to enhance patient motivation [18–23] in developing countries – and considering particularly its potential to improve TB treatment adherence and care [24–30] – we carried out a SMS-based intervention. The protocol of this trial was published [31] and registered (PACTR201307000583416).

## Methods

More details on the methodology of this study have been published [31]. There were no significant changes to the initial protocol. The findings of this study are reported according to CONSORT (Consolidated Standards of Reporting Trials) 2010 statement [32].

### Objectives

The primary objective was to evaluate the effectiveness of daily SMS reminders to increase the proportion of cure of adult patients who were newly diagnosed with sputum positive pulmonary tuberculosis (SS + PTB) and followed in Treatment and Diagnostic Centres (TDC) in Yaoundé, Cameroon. We formulated the hypothesis that sending daily SMS to remind patients to take their prescribed anti-tuberculosis medication – on top of selective DOT (once weekly or monthly distribution of drug) – would increases the proportion of patients cured from 65% (DOT, no SMS intervention) to 85% (DOT, with SMS intervention).

The secondary objective was to evaluate the effectiveness of sending SMS reminders on: patients’ self-estimation of adherence to anti-tuberculosis treatment; the attendance to appointments; the punctuality to appointment dates, and the degree of patients’ satisfaction at 6 months.

### Trial design

This was a randomised, single blinded (for healthcare professionals delivering the drugs and assessing sputum-smears), controlled and multicentre trial. Patients were randomly allocated to one of two groups in each centre (ratio 1:1): an intervention group in which participants received daily SMS reminders in addition to the usual treatment and a control group where participants did not receive reminders.

### Participants and setting

Patients were recruited in Treatment and Diagnostic Centres (TDCs) of Yaoundé between February and October 2013, and followed until April 2014. The city of Yaoundé has seventeen (17) TDCs, of which sixteen (16) were eligible, as the TDC located in Yaoundé prison was excluded (prisoners are not allowed to have phones).

Eligible patients had to be adults (> 18 years), newly diagnosed SS + PTB (clinical signs of pulmonary tuberculosis with two microscopic sputum smear examinations, one of which at least had to be positive), be able to read French or English, and in possession of a mobile phone for personal use. In addition, they had to know how to open and read an SMS on their telephone and to have given written consent to study participation after having been informed about it. After recruitment, all participants received a welcome SMS, followed by a phone call from the research team in order to check whether the phone number really belonged to the patient. This last step determined the definitive inclusion of the patient into the study.

### Interventions

All participants received the usual care (selective DOT) from the PNLT. This includes: free anti-tuberculosis treatment with an intensive phase (four-drug therapy for the first 2 months made of rifampicin, isoniazid, ethambutol and pyrazinamide) followed by a continuation phase (bi-therapy from the third to the sixth month made of rifampicin and isoniazid), appointments for drug refills (weekly for the first 2 months and monthly between the third and sixth months), three smear control tests at the end of the second, fifth and sixth months, and education coupled with counselling of patients throughout the process.

Patients in the intervention group received, in addition, free and daily SMS reminders in French (French being one of national languages, and all of them were French-speaking) throughout the 6 months of treatment. In the intervention group, the phone call made after the welcome SMS was not only to guarantee that the phone belonged to the patient but also that the latter had read the content of the welcome SMS. The SMS content was designed during a workshop by the research team. The purpose was to deliver simple and easy SMS aiming at reminding, inciting or motivating the patients to take the prescribed anti-tuberculosis medications. To maintain the patients’ attention and interest, these messages changed every 2 weeks. Here is the batch of SMS sent during the first 2 months of treatment as an illustration of this:

(i) “Hello, please don’t forget to take your TB treatment”; (ii) “Hello, it’s important to take your TB drugs every day”, (iii) “Hello, taking TB drugs daily increases your chance of healing”, (iv) “Hello, thank you for continuing to take TB treatment”, etc.). Additional encouraging messages were also sent every 2 weeks (e.g.: (i) “Congratulation, the second week of treatment is over!”, (ii) “Congratulation, the first month of treatment is over!”, (iii) “Congratulation, the sixth week of treatment is over!”, (iv) “Congratulation, the second month of treatment is over!”, etc.). Messages were neutral, similar for both genders. Patients also received a message at the end of treatment to thank them for participating in the study.

At each visit, health professionals verified (by asking to patient) that patients still had same telephone contacts they provided when they were included in the study. Patients in the control group only received a welcome SMS at the beginning, the phone call after the welcome SMS, and a SMS at the end of the 6 months treatment period to thank them for their participation. As such, they did not receive daily SMS reminders.

### Outcomes

The primary outcome was the proportion of patients cured. These were defined as such when having completed the 6 months treatment with bacteriologic confirmation of conversion to sputum smear-negative at 6 months. We also associated as primary outcome the proportion of patient with “treatment success”, defined as having completed the 6 months treatment and having negative sputum smears at 5 months.

The secondary outcomes were: (i) patients’ self-estimation of adherence to treatment on a 0 to 100% scale during the last 30 days before appointments, (ii) proportion of patient attending appointments (whether the patient was present or not), (iii) the punctuality to appointment dates (“early” for participants coming more than four days before the appointment date, “prompt” for participants coming between four days before and four days after the appointment date and “late” for participants coming more than four days after the appointment date). They were evaluated during follow-up visits at two, five, and six months. Finally (iv), we evaluated the degree of patients’ satisfaction at the end of the sixth month using Likert Scale.

During the study, patients had six mutually exclusive possible treatment outcomes: “cured” (sputum smear-negative at 6 months of treatment); “treatment success” (sputum smear-negative at 5 months of treatment but no final smear result at 6 months); “treatment failure” (sputum smear-positive at five or 6 months of treatment); “transferred” (patient referred in another TDC out of the study zone in order to continue treatment without feedback from the receiving TDC; “died” (patient died between the screening phase and the end of treatment, no matter the cause of death) and “loss to follow-up”.

### Sample size

The sample size calculation was based on an estimation of the baseline proportion of patients “cured” with DOTS strategy in Cameroon of 65% [17]. Our hypothesis was that the proportion of patients cured in the intervention group would increase to 85%. Thus, this study was designed to detect a 20% increase of cures, with 80% of power, 5% of alpha error and « two-sided » tests. Using online Open Epi software [33], the required sample size was 166 participants. Considering the risks of loss-to-follow-up and of missing data we increased the sample size to 260 eligible patients.

### Randomisation, allocation and blinding

Based on the activity of TDC (data obtained from PNLT), we calculated the proportion of patients to be recruited in each TDC (e.g. The Jamot Hospital which is the biggest TDC of Yaoundé was expected to contribute about 55% of the sample). Eligible and consenting patients attached to each centre received numerical codes (e.g. A001, A002, etc. for centre A). This code was assigned consecutively over the recruitment of patients, who were also stratified by recruitment centres (use of random block sizes), and randomised into the two groups (intervention and control groups) with an allocation ratio of 1:1. Randomisation was carried out by research team using a computer generated list. This allocation list was kept by the research team and concealed to healthcare professionals. Once a patient was recruited by a healthcare professional, the study identifiers and telephone numbers were communicated to the research assistant who allocated him/her in one of the two groups according to the randomisation scheme. Thus, healthcare professionals were blinded to randomisation and allocation of patients into two groups during the recruitment phase, thereby preventing allocation or selection biases.

### Data collection and supervision

Data on patients were collected by healthcare professionals taking care of them using patient follow-up forms elaborated by the research team (which contained all the necessary questionnaires, scales, etc.) and kept in the patients’ health records. These forms were updated during each visit (day of inclusion, second, fifth, and sixth months). An information and training session organized before the start of the study allowed training healthcare professionals in the correct completion of the forms.

A research assistant collected telephone numbers during the recruitment phase from the patient forms once a week at each TDC in order to continuously provide and update the phone numbers database with relevant phone numbers of newly enrolled patients. At the end of the 6th month, healthcare professionals administered a self-evaluation questionnaire to evaluate patient satisfaction.

Two sessions of supervision of research activities were carried out at the end of third and at eighth month after recruitment. The first session aimed to evaluate the recruitment of patients in each TDC, the completion of the consent form, the sending of SMS reminders as well as the checking of their reception by patients, and the quality of collected data in all TDC by healthcare professionals. The second session aimed to evaluate the follow-up of patients in the 12 recruiting TDC according to research protocol and the quality of collected data. This supervision was made by an evaluator from the research team. At the end of each supervision activity, recommendations were made by the evaluator in order to correct observed discrepancies between the protocol and the findings on the ground.

### Statistical analysis

A data entry form was developed with the Epi Data Entry software (version 3.1, available freely at http://www.epidata.dk/) was used to enter participants’ data and to carry out double data entry and validation. Outcomes (primary and secondary) were analysed on an « Intention to Treat » (ITT) basis. For primary outcomes, all patients lacking information on their smear test at 5 or 6 months were considered as not cured (worst case scenario).

Results of this study are presented as mean (standard deviation) and median (inter-quartile ranges) for continuous variables depending on their underlying distribution, and frequencies (percentages) for categorical variables. The two groups are described according to their baseline characteristics and primary and secondary outcomes at 2, 5, and 6 months. Differences between the two groups were tested using Student (T-test) and Mann-Whitney tests for continuous variables according to the underlying variable distribution; Chi-square and Fisher’s exact tests for categorical or nominal variables. The comparisons are reported as odds ratios (OR) with 95% confidence intervals (CI).

### Additional analyses

A logistic regression model was built to identify sociodemographic factors (age, gender, level of education, socio-economic category, marital status, incomes and HIV status) that could help to predict which patients were likely to be lost-to follow-up during their treatment. Data from this study were analysed using SPSS programme (v22.0) and Online Open Epi Software [33].

### Material and equipment for sending SMS

A web proprietary platform [34] has been used to send daily SMS messages to participants. This application can log SMS messages and allows to produce reports of those sent. The platform was tested before using it for the intervention to ensure its effectiveness (failure rate), especially in sending grouped SMS. This system proved to be effective and satisfactory.

## Results

### Recruitment and patient flow

Patients were recruited in 12 of the 16 eligible TDCs of Yaoundé city only. Two hundred eighty-two (282) potentially eligible patients were contacted between February and October 2013 for their recruitment in the study, of whom three declined to participate. Two hundred seventy-nine (279) patients were thus randomly allocated to two groups (137 to the intervention group and 142 to the control group) and were followed during 6 months.

At 2 months, 125 (91.2%) patients in intervention and 124 (87.3%) in the control groups were still followed (OR = 1.51 [0.69, 3.27]; *p* = 0.29). At 5 months, they were 112 (81.7%) and 108 (76.0%) respectively (OR = 1.41 [0.78, 2.51]; *p* = 0.24). Finally at 6 months, they were 88 (64.2%) and 90 (63.4%) respectively (OR = 1.03 [0.63, 1.69]; *p* = 0.88).

**Fig. 1:**
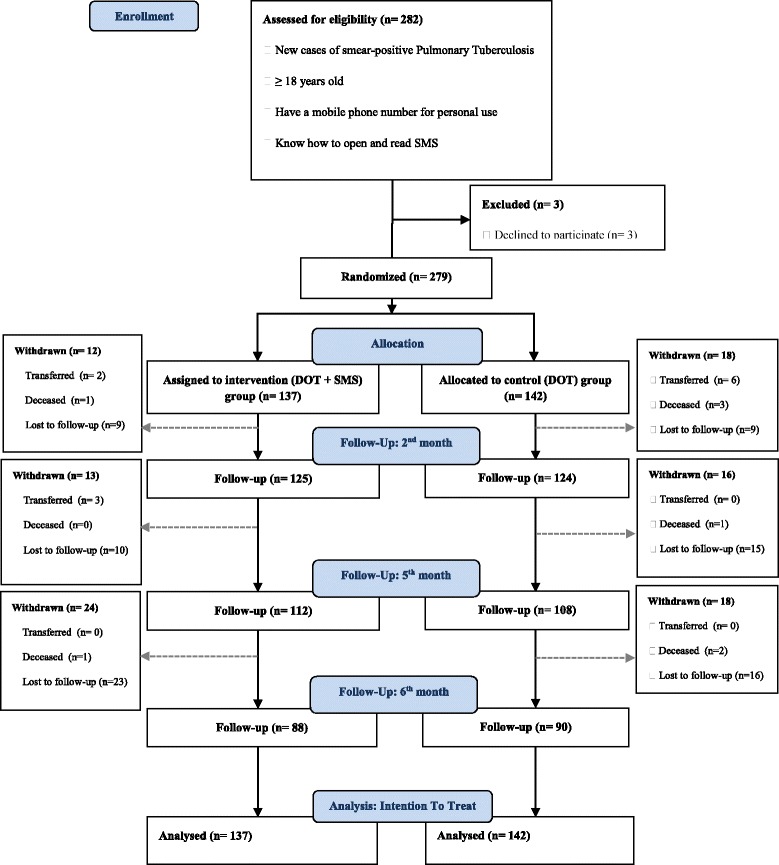
Flow Diagram of study design

Approximately half (*n* = 24) of patients withdrew in the intervention group between the fifth and the sixth month, while in the control group, about one-third (*n* = 18) of patients withdrew at each appointment. Figure 1 shows the flow diagram of patients throughout the study.

### Baseline data

Data from 279 randomized participants were analysed. There is a homogeneous distribution of participants in both groups (Table 1), except for the prevalence of TB - HIV co-infection: about 20% of patients were HIV positive in the intervention group, less than 10% in the control group (*p* = 0.02).

**Table 1:** Demographics and baseline characteristics of participants in both groups

Variables	Intervention group (DOT + SMS) *n* = 137	Control group (DOT) *n* = 142
Gender: n (%)		
Male	86 (62.8)	79 (55.6)
Female	51 (37.2)	63 (44.4)
Age (years): n (%)		
18–25	22 (16.1)	34 (23.9)
26–40	84 (61.3)	79 (55,7)
41–55	26 (18.9)	20 (14,1)
56–80	5 (3.7)	9 (6,3)
Level of Education: n (%)		
None	7 (5.2)	4 (2.9)
Nusery/primary	38 (27.9)	34 (23.9)
Secondary	69 (50.1)	74 (52.1
Tertiary	23 (16.8)	30 (21.1)
Marital Status: n (%)		
Single	84 (61.3)	93 (65.5)
Married	40 (29.1)	37 (26.0)
Cohabitation	11 (8.0)	7 (5.0)
Widower	1 (0.8)	5 (3.5)
Divorced	1 (0.8)	0 (0.0)
Social Category: n (%)		
Student	21 (15.3)	25 (17.6)
Working in public sector	7 (5.1)	14 (9.8)
Working in formal private sector	20 (14.6)	15 (10.6)
Working in informal private sector	41 (30.0)	37 (26.0)
No work	48 (35.0)	51 (36.0)
Monthly Income (XAF): n (%)		
< 50,000	40 (29.2)	31 (21.8)
50,000–149,000	20 (14.6)	25 (17.6)
> 149,000	5 (3.7)	3 (2.1)
Not reported (missing)	72 (52.5)	83 (58.5)
HIV Status: n (%)		
Negative	85 (62.0)	98 (69.0)
Positive	27 (19.7)	12 (8.5)
Unknown	25 (18.3)	32 (22.5)
Patient on ART (yes): n (%)	10 (7.3)	7 (4.9)
Microscopy (positive): n (%)	137 (100)	142 (100)
Treatment plan (2RHEZ/4RH): n (%)	137 (100)	142 (100)
Duration of treatment (months): Mean (SD) / Median (Q1-Q3)	6.6 (1.2) / 6.2 (6.0–6.7)	6.5 (1.3) / 6.1 (6.0–6.6)

*DOT* directly observed treatment, *SMS* short message service, *1 US Dollar* 599 XAF (F CFA, currency used in Cameroon), *HIV* human immunodeficiency virus, *ART* anti-retroviral therapy, *2RHEZ/4RH* 2 months of rifampicin, isoniazid, ethambutol, pyrazinamide and 4 months of rifampicin and isoniazid, *SD* standard deviation, *Q1* first quartile, *Q3* third quartile

### Outcomes and estimation

#### Primary outcomes

At the 5th month, the treatment success (Table 2) was 81% (*n* = 111) in the intervention group and 74.6% (*n* = 106) in the control group (OR = 1.45 [0.81, 2.56]; *p* = 0.20). About 1.5% (*n* = 2) of the patients had a treatment failure (patients with still sputum smear-positive at 5 months) in each group. One of the two sputum smear-positive patients in the intervention group was transferred between the second and fifth month.

**Table 2:** Primary outcomes

Primary outcomes	*Intervention group* *(DOT + SMS) n = 137*	*Control group* *(DOT)* *n = 142*	*Effect Estimate*
	n (%)	n (%)	*OR [95% CI]; p*
Microscopy at 5 months			
Negative (successfully treated)	111 (81.0)	106 (74.6)	1.45 [0.81, 2.56]; 0.20
Microscopy at 6 months			
Negative (cured)	87 (63.5)	88 (62)	1.06 [0.65, 1.73); 0.79

*DOT* directly observed treatment, *SMS* short message service, *OR* odds ratio, *CI* confidence interval

At month six, the proportion of patients cured (Table 2) was 63.5% (*n* = 87) in the intervention group and 62% (*n* = 88) in the control group (OR = 1.06 [0.65, 1.73]; *p* = 0.79). One patient in the intervention group and two in the control group were still sputum smear-positive at six months.

#### Secondary outcomes

There were no significant differences between the two groups in any secondary outcomes as shown in Table 3. It should be noted that strong adherence to treatment during the last 30 days of the three appointments – as described by patients using visual analogue scale – was observed in both groups.

**Table 3:** Secondary outcomes

Secondary outcomes	Intervention group (DOT + SMS) n = 137	Control group (DOT) n = 142	Effect Estimate
	n (%)	n (%)	*OR [95% CI]; p*
At 2 months			
Attendance to appointments			
Number of patients followed	125 (91.2)	124 (87.3)	1.51 [0.69, 3.27]; 0.29
Number of patients withdrawn	12 (8.7)	18 (12.7)	0.66 [0.30, 1.43]; 0.29
Transferred out	2 (1.5)	6 (4.2)	0.33 [0.06, 1.69]; 0.16
Deceased	1 (0.7)	3 (2.1)	0.34 [0.03, 3.31]; 0.33
Lost to follow-up	9 (6.6)	9 (6.4)	1.03 [0.39, 2.70]; 0.93
Punctuality to appointment dates			
Early and Prompt	98 (71.5)	104 (73.2)	0.91 [0.54, 1.55]; 0.74
Late	27 (19.7)	21 (14.8)	1.41 [0.75, 2.64]; 0.27
	Mean (SD) / (Min, Max)	Mean (SD) / (Min, Max)	MD *[95% CI]; p*
Adherence to prescription drugs (%, VAS)	98.5 (6.3) / (50, 100)	99.3 (3.5) / (70, 100)	- 0.8 [−2.0, 0.5]; 0.25
At 5 months			
Attendance to appointments			
Number of patients followed	112 (81.7)	108 (76.0)	1.41 [0.78, 2.51]; 0.24
Number of patients withdrawn	25 (18.3)	34 (24.0)	0.70 [0.39, 1.26]; 0.24
Transferred out	5 (3.7)	6 (4.2)	0.85 [0.25, 2.88]; 0.80
Deceased	1 (0.7)	4 (2.8)	0.25 [0.02, 2.29]; 0.18
Lost to follow-up	19 (13.9)	24 (17.0)	0.79 [0.41, 1.52]; 0.48
Punctuality to appointment dates			
Early and Prompt	69 (50.4)	70 (49.3)	1.04 [0.65, 1.66]; 0.85
Late	44 (32.1)	38 (26.8)	1.29 [0.77, 2.17]; 0.32
	Mean (SD) / (Min, Max)		
Adherence to prescription drugs (%, VAS)	98.9 (5.8) / (50, 100)	99.2 (3.8) / (70, 100)	- 0.3 [−1.5, 1.1]; 0.73
At 6 monthss			
Attendance to appointments			
Number of patients followed	88 (64.2)	90 (63.4)	1.03 [0.63, 1.69];0.88
Number of patients withdrawn	49 (35,8)	52 (36.6)	0.96 [0.59, 1.57]; 0.88
Transferred out	5 (3.6)	6 (4.2)	0.85 [0.25, 2.88]; 0.80
Deceased	2 (1.5)	6 (4.2)	0.33 [0.06, 1.69]; 0.16
Lost to follow-up	42 (30.7)	40 (28.2)	1.12 [0.67, 1.88]; 0.64
Punctuality to appointment dates			
Early and Prompt	65 (47.4)	69 (48,6)	0.95 [0.59, 1.52]; 0.84
Late	23 (16.8)	21 (14.8)	1.16 [0.61, 2.21]; 0.64
	Mean (SD) / (Min, Max)	Mean (SD) / (Min, Max)	MD *[95% CI]; p*
Adherence to prescription drugs (%, VAS)	99.7 (1.8) / (80, 100)	99.5 (1.7) / (90, 100)	0.2 [−0.4, 0.7]; 0.53

*DOT directly observed treatment, SMS* short message service, *OR* odds ratio, *CI* confidence interval, *Early* at least 5 days before, *Normal* between 4 days before and 4 days after, *Late* starting from 5 days after, *SD* standard deviation, *MD* mean difference, *VAS* visual analog scale

Finally, we found a very high level of and similar satisfaction in both groups, regarding (i) general management of patients (99.5% of satisfaction in the intervention and 99.2% in the control group (MD: 0.3% [− 0.4, 1.0%]; *p* = 0.41) and (ii) support provided for adherence to drug prescriptions (99.6% of satisfaction in the intervention group and 99.1% in the control group (MD: 0.5% [− 0.2, 1.2]; *p* = 0.17).

### Additional analyses

Furthermore, our study confirms that patients who are retained during the 6 months in DOT such as practiced currently – both in intervention and control groups respectively – have a very high cure proportion at 6 months, if the number of patients with smear-negative is divided by the number of patients present at the last appointment for each group (per protocol analysis): 98.9 and 97.8% (OR = 1.97 [0.17, 22.2]; *p* = 0.73).

As we observed a high rate of withdrawn patients throughout the study (lost to follow-up or transferred participants for whom no information about outcome is available) and especially before the appointment of six months (34.3%; *n* = 47 in the intervention group and 32.4%; *n* = 46 in the control group), we examined and compared the sociodemographic profile of these withdrawn patients among the 2 groups (Table 4). Apart from HIV status (as observed in the baseline), there was no significant difference between both groups. In addition, we also examined and compared the sociodemographic profile of these withdrawn patients with the patients that remained in the trial throughout (Table 5). The binary logistic regression test performed was unable to identify any sociodemographic characteristics predicting lost-to-follow-up.

**Table 4:** Sociodemographic characteristics of withdrawn participants (transferred and lost to follow-up) at 6 months in intervention group versus control group

Variables	Intervention group (DOT + SMS) *n* = 47	Control group (DOT) n = 46	p
Gender: n (%)			
Male	33 (70.2)	26 (56.5)	0.17
Age (years): n (%)			
18–25	10 (21.3)	8 (17.4)	0.13
26–40	28 (59.6)	26 (56.5)	
41–55	9 (19.1)	7 (15.2)	
56–80	0 (0.0)	5 (10.9)	
Level of Education: n (%)			
None	3 (6.4)	1 (2.2)	0.65
Nusery/primary	16 (34)	16 (34.8)	
Secondary	23 (48.9)	21 (45.7)	
Tertiary	5 (10.6)	8 (17.4)	
Marital Status: n (%)			
Single	32 (68.1)	28 (60.9)	0.62
Married	12 (25.5)	14 (30.4)	
Cohabitation	2 (4.3)	1 (2.2)	
Widower	1 (2.1)	3 (6.5)	
Social Category: n (%)			
Student	5 (10.6)	6 (13.0)	0.54
Working in public sector	1 (2.1)	3 (6.5)	
Working in formal private sector	9 (19.1)	6 (13.0)	
Working in informal private sector	15 (31.9)	10 (21.7)	
No work	17 (36.2)	21 (45.7)	
Monthly income (XAF): n (%)			
< 50,000	18 (38.3)	10 (21.7)	0.21
50,000–149,000	5 (10.6)	8 (17.4)	
> 149,000	1 (2.1)	0 (0.0)	
Not reported (missing)	23 (48.9)	28 (60.9)	
HIV Status: n (%)			
Negative	32 (68.1)	31 (67.4)	0.03
Positive	11 (23.4)	4 (8.7)	
Unknown	4 (8.5)	11 (23.9)	
Patient on ART (yes): n (%)	2 (4.3)	3 (6.5)	0.62

*DOT* directly observed treatment, *SMS* short message service, *1 US Dollar* 599 XAF (F CFA, currency used in Cameroon), *HIV* human immunodeficiency virus

**Table 5:** Sociodemographic characteristics of withdrawn participants (transferred and lost to follow-up) versus all followed participants at 6 months

Variables	Withdrawn at 6th month (Transferred out + lost to follow-up) *n* = 93	Present or deceased at 6th month *n* = 186	p
Gender: n (%)			
Female	34 (36.6)	80 (43.0)	0.30
Male	59 (63.4)	106 (57.0)	
Age (years): n (%)			
18–25	18 (19.4)	38 (20.4)	0.99
26–40	54 (58.1)	109 (58.6)	
41–55	16 (17.2)	30 (16.1)	
56–80	5 (5.4)	9 (4.8)	
Level of education: n (%)			
None	4 (4.3)	7 (3.8)	0.09
Nusery/primary	32 (34.4)	40 (21.5)	
Secondary	44 (47.3)	99 (53.2)	
Tertiary	13 (14.0)	40 (21.5)	
Marital status: n (%)			
Single	60 (64.5)	117 (62.9)	0.21
Married	26 (28.0)	51 (27.4)	
Cohabitation	3 (3.2)	15 (8.1)	
Widower	4 (4.3)	2 (1.1)	
Divorced	0 (0.0)	1 (0.5)	
Social category: n (%)			
Student	11 (11.8)	35 (18.8)	0.17
Working in public sector	4 (4.3)	17 (9.1)	
Working in formal private sector	15 (16.1)	20 (10.8)	
Working in informal private sector	25 (26.9)	53 (28.5)	
No work	38 (40.9)	61 (32.8)	
Monthly income (XAF/ US Dollars): n (%)			
< 50,000	28 (30.1)	43 (23.1)	0.36
50,000–149,000	13 (14.0)	32 (17.2)	
> 149,000	1 (1.1)	7 (3.8)	
Not reported	51 (54.8)	104 (55.9)	
HIV status: n (%)			
Negative	63 (67.8)	120 (64.5)	0.40
Positive	15 (16.1)	24 (12.9)	
Unknown	15 (16.1)	42 (22.6)	

Step by step binary logistic regression was done for all variables (*p* > 0.05)

### Limitations

Only 12 of the 16 TDC of Yaoundé participated in this study. Two elements can explain the non-recruitment of patients in these TDCs: the refusal of some health professionals to participate in the study and staff rotations that occurred just after the beginning of the study.

In addition, some TDCs have not achieved the quotas of recruitment that were set for them quickly enough. Thereby, this led the extension of the time needed to recruit patients (10 months instead of 4 months as initially planned).

Another problem concerned the collection of some data in TDC (namely participants’ phone numbers) in order to constantly update the SMS distribution list. The initial planned collection frequency was twice a week during the recruitment phase, but for logistical reasons it was reduced to once a week.

As illustrated in the flow diagram of patients, we observed a significant number of total withdrawn patients (lost to follow-up, transferred and deceased) throughout the study. At the appointment of 6 months, the total of withdrawn patients was 35.8% (*n* = 49) in the intervention group and 36.6% (*n* = 52) in the control group (OR = 0.96 [0.59–1.57]; *p* = 0.88).

Finally, it appeared impossible to assess drug resistance to patients with failure at 5 months of treatment with the GeneXpert [35] as announced in the research protocol. The functional capabilities of PNLT at the time of the study did not allow to carry out this exam systematically. The contacts of these four participants affected were given to the national program coordinator to take them in charge outside of the study.

## Discussion

This study explores the potential of a m-health intervention to improve the proportion of pulmonary tuberculosis patients cured in Cameroon. The cure rate was of 65% of the treated patients at the beginning of the study, and we expected to improve it to 85%, corresponding to the WHO recommendations [36]. Many national programs have introduced weekly or monthly observed treatment strategies, which do no longer correspond to original DOT which recommend to healthcare professionals a daily follow-up of patients for drug intake, especially in the intensive phase. In this context, sending daily SMS reminders requires less resources as daily DOT, but also less time, staff, organization, and funding [37, 38] and prevents stigmatization of patients.

Unfortunately, the results obtained in this study suggest that the proportion of patients cured at 6 months is almost similar in both groups (around 63%) and below the 85% expected.

Several reasons explain our results. Firstly, we used an intention to treat analysis, which considers as “treatment failure” any patient missing the final appointment, without giving precise information to explain his/her absence. This contributes to the very low proportion of the patients cured. Most of patients who dropped-out – or were transferred – did so between the 5th and 6th month. We may presume that many of them were in fact cured, felt well, and were not willing to pay out of their pocket for the last required sputum exam for not being part of the free treatment package offered by the national TB control program. Secondly, one of the main public health objectives in the management of SS + PTB patients is to reduce transmission throughout the population. As such, international guidelines require at least 6 months treatment regimens even if it is known that patients who had TB once are at higher risk of reinfection than subjects who never experienced TB-disease in their life before [39]. Is this risk really higher for newly diagnosed SS + PTB patients who fulfilled the TB treatment protocol – 2 months of a quadritherapy with Rifampicin, Isoniazid, Ethambutol, and Pyrazinamide followed by 4 months of bi-therapy with Rifampicin and Isoniazid – and proved smear-negative sputum after 5 months? Unfortunately we did not find conclusive statements in the medical literature.

The results obtained at 5 months, showed that the proportion of patients with treatment success is higher (approximately 6% difference) in the intervention group compared to the control group (81% versus 74.6%, OR = 1.45 [0.81, 2.56]; *p* = 0.20). Although not statistically significant, it suggests a trend in favour of SMS reminders, with cure rates approaching what is expected from WHO. This is consistent with a study carried out in the Republic of South Africa which found a treatment success of 72.94% in the SMS group compared to 69.4% in the DOT group [27].

The use of mHealth in general – and SMS for the management of TB – has been described in many studies [24–30]. For improving TB treatment adherence and cure proportion, a systematic review conducted in 2013 noted the lack of sufficient evidence on the effectiveness of SMS [29]. In our study, despite acceptability and positive perception of patients about SMS reminders [22], the relative weak added effect of SMS to support treatment adherence and to improve cure and treatment success rates of SS + PTB patients at 5 and 6 months respectively, led us to ask questions about the relevance and challenges of such intervention in low and middle income countries like Cameroon. Should SMS reminders be formatted differently in order to contribute to better treatment adherence? If more patients are retained in DOT, then more of them will have their cured status confirmed and resistant cases will be prevented ultimately [37].

Here below, we discuss some lessons learned during this study and propose avenues that may improve the effectiveness of a mHealth intervention in general (and by SMS). This is so in particular to sustain the existing DOT, reduced in reality from “daily” to “weekly” or even fewer directly observed strategies. These suggestions are an addition to this study [19] and highlights steps, processes and considerations to take into account in order to develop an SMS-based intervention for promoting adherence to TB treatment.

### Further avenues and suggestions

#### Training of health professionals to improve patient education

The implementation of a mHealth intervention to support DOT needs to train health professionals to better understand challenges like patient education in order to enable them to manage their own treatment course [40, 41]. Principles and concepts underlying effective patient education require the health staff to open a dialogue where the patient is given space and time to become aware about her/his preferences, her/his constraints, and to express these to the care-giver.

#### Customized messages and management of the patient profile

Another point of the m-health intervention is to explore customized motivational messages in relation to the patient’s profile at the beginning, with a special attention towards the end of the treatment where patients are almost free to free of symptoms. The motivational messages would target some features of the compliance with treatment: to invite, to motivate, to encourage and to recall appointment date, or even to reward accomplished steps of the cure. Furthermore, the SMS contents should take into account the information-motivation-behavioral (IMB) skills model including contextual elements such as culture, language, beliefs, and factors influencing access to health care [19, 42, 43].

In our setting, withdrawn population is mainly composed of people who are: males; aged between 26 and 40 years; with nursery, primary or secondary level of education; single; not working or working in the informal private sector, and with no or low monthly income. Although the association is not statistically significant, the implementation of an effective information system – using the potential of information technology as recommended by WHO [44] for collecting profile and treatment data – could be effective for improving compliance.

#### Interactive mHealth intervention

Our study corroborated the results of a study exploring the acceptance and perception of SMS uses to improve TB treatment compliance [22]. However, sending only SMS denotes a “one-way” communication which is contradictory to effective patient education. We therefore suggest to develop an interactive m-health combining SMS reminders, sent to patients by the healthcare staff, with the possibility given to patients to submit their own questions and concerns back to the healthcare professionals. In addition, phone-calls completing SMS messages could also reinforce therapeutic relationship enabling patients to respond treatment requirements as for example to come back for final appointment with smear test at 6th month. As our results suggest, the mHealth interventions should be especially targeted at maintaining patient compliance between months five and six, where around 50% of patient’s withdrawn are registered. We postulate that it is relevant to emphasise and increase interactions with patients during this period. The challenge also lies in the health staff, which should be able to deliver a high quality and efficient patient-centered relationship.

Beyond the motivational messages, mHealth-based interventions which would try to reproduce the DOT principle, namely the observation of patient taking his medication, could be an alternative. As the coverage of smartphones expands, one could imagine that patients start to send footages of either themselves taking their medications, or the packaging of drugs on a daily basis (“picture-observed drug intake”). Alternatively, we can explore mHealth approaches that rely on social incentives, such as cooperation and competition, to motivate users in achieving health goals [45].

#### Adaptation of health policy with concern to financing TB care

The monitoring of the cure proportion is a crucial indicator of any TB care program. Therefore, patients should be encouraged for the final clinical and laboratory exams at 6 months. However, our data with important drop-out rate after 5 months of treatment indicate that the actual procedures – requiring out-of-the-pocket payment by the patient to afford this final exam – could represent a major obstacle to this. A national TB control program should include at least this final exam, as the Global Fund [46] provides TB drugs free of charge for patients. The fact that socially underprivileged people are more vulnerable to become infected with TB [47] underlines the relevance to adapt health policy based on real costs of TB care. Taking into account the social context of patients will enable to optimise patient retention, enhance DOTS performances and reinforce the reliability of national epidemiological surveillance.

## Conclusions

This study suggests that SMS reminders do not statistically significantly increase proportions of the treatment success at 5 months and the cure at 6 months. However, the low proportion of patients cured at 6 months may be an underestimation due to a high dropout rate between the fifth and the sixth month of treatment. We hope that lessons learned from this study and outlined above will help to reduce the dropout rate and increase the effectiveness of future mHealth intervention studies about management of tuberculosis patients.

## Abbreviations

2RHEZ/4RH: 2 months of Rifampicin, Isoniazid, Ethambutol, Pyrazinamide and 4 months of Rifampicin and Isoniazid
ART: Anti-retroviral therapy
ARV: Antiretroviral
BCG: Bacille Calmette Guerin
CONSORT: CONsolidated Standards of Reporting Trials
DOTS: Directly observed treatment short-course
HIV: Human immunodeficiency virus
ISPM: Institute of Social and Preventive Medicine
ITT: Intention to treat
KB: Koch Bacillus
Max: Maximum
MD: Mean difference
mHealth: Mobile Health
Min: Minimum
OR: Odds ratio
PLWHIV: Persons living with HIV
PNLT: National Program for the fight against tuberculosis
SD: Standard deviation
SMS: Short message service
SS + PTB: Sputum Smear-positive Pulmonary Tuberculosis
TB: Tuberculosis
TDC: Treatment and diagnostic centres
VAS: Visual analog scale
WHO: World Health Organisation
